# Emerging SARS-CoV-2 Genotypes Show Different Replication Patterns in Human Pulmonary and Intestinal Epithelial Cells

**DOI:** 10.3390/v14010023

**Published:** 2021-12-23

**Authors:** Gabriel Augusto Pires de Souza, Marion Le Bideau, Celine Boschi, Lorène Ferreira, Nathalie Wurtz, Christian Devaux, Philippe Colson, Bernard La Scola

**Affiliations:** 1 Unité de Recherche Microbe Phylogeny and Evoluition (MEPHI), Institut de Recherche pour le Développement (IRD), Assistance Publique—Hôpitaux de Marseille (AP-HM), Aix-Marseille Université, 27 Boulevard Jean Moulin, 13005 Marseille, France; gabriel-augusto.PIRES-DE-SOUZA@etu.univ-amu.fr (G.A.P.d.S.); Marion.LE-BIDEAU@ap-hm.fr (M.L.B.); Celine.BOSCHI@ap-hm.fr (C.B.); lorene.FERREIRA@univ-amu.fr (L.F.); nathalie.wurtz@univ-amu.fr (N.W.); christian.devaux@mediterranee-infection.com (C.D.); philippe.COLSON@univ-amu.fr (P.C.); 2 IHU Méditerranée Infection, 19-21 Boulevard Jean Moulin, 13005 Marseille, France

**Keywords:** SARS-CoV-2, COVID-19, viral culture, isolate, genotype, variants, Vero E6 cells, Caco-2 cells, Calu-3 cells

## Abstract

Severe Acute Respiratory Syndrome Coronavirus 2 (SARS-CoV-2) quickly spread worldwide following its emergence in Wuhan, China, and hit pandemic levels. Its tremendous incidence favoured the emergence of viral variants. The current genome diversity of SARS-CoV-2 has a clear impact on epidemiology and clinical practice, especially regarding transmission rates and the effectiveness of vaccines. In this study, we evaluated the replication of different SARS-CoV-2 isolates representing different virus genotypes which have been isolated throughout the pandemic. We used three distinct cell lines, including Vero E6 cells originating from monkeys; Caco-2 cells, an intestinal epithelium cell line originating from humans; and Calu-3 cells, a pulmonary epithelium cell line also originating from humans. We used RT-qPCR to replicate different SARS-CoV-2 genotypes by quantifying the virus released in the culture supernatant of infected cells. We found that the different viral isolates replicate similarly in Caco-2 cells, but show very different replicative capacities in Calu-3 cells. This was especially highlighted for the lineages B.1.1.7, B.1.351 and P.1, which are considered to be variants of concern. These results underscore the importance of the evaluation and characterisation of each SARS-CoV-2 isolate in order to establish the replication patterns before performing tests, and of the consideration of the ideal SARS-CoV-2 genotype–cell type pair for each assay.

## 1. Introduction

A newly-emerging coronavirus that infects humans was reported in December 2019 in patients presenting with viral pneumonia in Wuhan, China [1]. Coronaviruses are enveloped viruses, with a positive single-stranded RNA genome and various host animals [2]. They are divided into four genera: alpha, beta, gamma, and delta [3]. However, only alpha and beta coronaviruses are known to infect humans, leading to pathologies ranging from symptoms typical of the common cold to life-threatening respiratory diseases in the lower respiratory tract [3]. Before this new coronavirus was identified in China, only six human pathogenic coronaviruses were known [2].

The aetiologic agent of the outbreak in Wuhan was later identified as a beta-coronavirus, named Severe Acute Respiratory Syndrome Coronavirus 2 (SARS-CoV-2) [4,5], and was notable for its rapid spread. SARS-CoV-2 became a threat to global public health, as it represents a risk of the health systems in each country collapsing [6]. In March 2020, the World Health Organization (WHO) declared the new coronavirus disease, Coronavirus Disease 2019 (COVID-19), to be a pandemic [3]. Currently, COVID-19 has led to almost 195 million confirmed cases, and more than 4.2 million deaths had been reported worldwide on 30 July 2021 [6].

As efforts were made to contain the virus, the eyes of the scientific community turned to SARS-CoV-2. However, the high circulation of the virus favoured the emergence of viral variants, which have become predominant in some regions [7,8,9]. The emergence of new SARS-CoV-2 variants throughout the COVID-19 pandemic brought with them public health concerns, due to the increase in transmissibility [10,11,12] and especially regarding the effectiveness of vaccines [13,14], as mutations in the spike protein, which is a target for many of the approved vaccines, were observed [11,15,16].

Mutations at sites such as the amino-terminal domain (NTD) and the receptor-binding domain (RBD) have been associated with direct implications for virus infection rates due to the greater affinity of RBD to the angiotensin-2 converting enzyme (ACE2), which is known as the main receptor for SARS-CoV-2 [15]. Other evidence points to the selection of variants with greater virulence and resistance to the action of neutralising antibodies from convalescent or immunised individuals [10,11,12,15,16,17]. Viruses harbouring mutations that confer a competitive advantage regarding viral replication, transmission, or immunity escape will increase in frequency, becoming dominant variants [10,11,12,18].

The current genomic diversity of SARS-CoV-2 has a clear impact on viral epidemiology and virus-associated clinical practice, especially regarding transmission rates and the effectiveness of vaccines [19,20,21,22,23]. One challenging question of basic virology is about the extrapolation of data obtained from the SARS-CoV-2 virus which was responsible for the first wave of the pandemic in early 2020, and which was a close relative of the original Wuhan-Hu-1 isolate, to new SARS-CoV-2 variants, which exhibit substantial genetic and amino acid differences, especially regarding the standardisation of in vitro assays. As new variants emerge, cell culture models help characterise their cell tropism and virus replication kinetics, as well as the profiles of the induced cell damage [2,24,25,26,27]. These variants present a challenge in dealing with the COVID-19 pandemic.

In this study, we explore the replication of different SARS-CoV-2 isolates in three different cell lines which are known to be permissive to SARS-CoV-2: two human-derived cells, Caco-2 (intestinal epithelium) and Calu-3 (pulmonary epithelium), and one monkey renal epithelium-derived cell (Vero E6). The viruses were isolated in Marseille, France, during different waves of the pandemic. These isolated viruses include variants which have been classified as variants of interest or of concern by the Centers for Disease Control and Prevention (CDC) [28]. The results demonstrate the difficulties of establishing standards of virus replication and tropism for SARS-CoV-2, especially for in vitro assays, with its current diversity of genotypes.

## 2. Materials and Methods

### 2.1. Cell Line Culture

The Vero E6 cell line (American type culture collection ATCC^®^ CRL-1586™) was cultured in Minimum Essential Medium (MEM. Gibco, Thermo Fischer) containing 4% foetal bovine serum (FBS. Invitrogen) and 1% L-glutamine (L-GLn. Invitrogen) at 37 °C in a 5% CO_2_ atmosphere using 175 cm^2^ flasks. Every three days, the medium was replenished, and the confluent culture flask was subcultured by trypsinisation. Calu-3 cells (ATCC^®^ HTB-55™) were cultured in MEM containing 10% FBS and 1% L-Gln in 175 cm^2^ flasks. Caco-2 cells (ATCC^®^ HTB-37™) were also cultured in 175 cm^2^ flasks at 37 °C and 5% CO_2_, using Dulbecco’s Modified Eagle’s Medium (DMEM) supplemented with 10% FBS, 1% L-Gln, and 1% amino acids (Aa). In the assays using culture plaques, the Calu-3 and Caco-2 cells were prepared three days beforehand, and the Vero E6 cells one day beforehand; all of the cells were incubated at 37 °C and 5% CO_2_. 

### 2.2. Production of Fresh SARS-CoV-2 Inoculum

Vero E6 cell 12-well plates were prepared with 5 × 10^5^ cells/well, and cultured with 2 mL of 10% SFB MEM and 1% L-Gln incubated overnight at 37 °C in a 5% CO_2_ atmosphere. The fresh viral inoculum was produced from frozen stocks of 32 distinct isolated SARS-CoV-2 samples previously genotyped by whole-genome next-generation sequencing (Table S1) and thawed at room temperature. The supernatant from the previous day was removed and replaced with fresh medium. The inoculum of 5300 µL of the virus was then added to the well. The plaque was incubated again at 37 °C and 5% CO_2_ overnight. After incubation, the cell layer and the supernatant were collected and filtered at 0.2 µm, and 100 µL of the filtrates were collected for RNA extraction using the QIAamp 96 Virus QIAcube HT Kit (QIAGEN, Hilden, Germany) on the QIAcube HT System (QIAGEN, Hilden, Germany). RT-PCR was performed using the SuperScript III Platinum One-Step qRT-PCR Kit (Invitrogen, Carlsbad, EUA) in the Roche LightCycler^®^ 480 Instrument II. The primers were designed against the N gene (Fwd: 5′ GACCCCAAAATCAGCGAAAT 3′; Rev: 5′ TCTGGTTACTGCCAGTTGAATCTG 3′ and probe: 5′ FAM-ACCCCGCATTACGTTTGGTGGACC 3′). After the RT-qPCR, the supernatants which collected 24 h.p.i. from Vero E6 infected cells were diluted based on the RT-qPCR to an specific Ct (Ct = 20), and TCID50 was performed in the Vero E6 cells using nine isolates, four replicate wells by dilution, performed in duplicate, and read 7 days post-infection. The TCID50 was calculated according to the Spearman and Kärber algorithm.

### 2.3. Cell Infection and Assessment of SARS-CoV-2 Replication 

The 24-well plates were previously prepared with 4 × 10^5^ cells/well of Caco-2 and Calu-3, and with 2 × 10^5^ cells/well of Vero E6, grown in 1 mL of their corresponding medium, as mentioned above. For the Calu-3 and Caco-2 cells, the medium was removed and replaced with new medium the previous day. The cells were kept incubated at 37 °C and 5% CO_2_ until infection, immediately after the PCR. Before infection, one well of each plate was trypsinized, and the cells were counted in a disposable neubauer chamber to determine the multiplicity of infection (MOI). Fresh SARS-CoV-2 inocula were diluted in MEM 4% SFB and 1% L-Gln for a Cycle Threshold (Ct) value of 20. After dilution, 200 µL of the inoculum was added to the corresponding well in the plate. The adsorption was performed by centrifugation at 2272× *g*, for 1 h at 37 °C (Sorvall Legend XT/XF, M-20 rotor, Thermo Scientific™,Waltham, MA, USA, 75217406/DEL). After the adsorption, the medium was discarded, and the wells were washed twice with 1 mL of the medium. The medium was replaced (1 mL) and 100 µL was collected for the PCR, with this being considered time = 0 (T0). For the Caco-2 and Calu-3 cells, 100 µL of the supernatant was also collected 1 (t1), 3 (t3), and 7 (t7) days post-infection (d.p.i.). The final volume of 1 mL was maintained throughout the manipulation. For Vero E6, aliquots were collected on days 1 and 3 p.i. The supernatants were stored at −80 °C until the RNA extraction was performed, as well as the PCR, as described above. Replication was assessed based on the ΔCt of the samples where ΔCt = Ct_t0_ – Ct_t3_. Three different experimenters carried out this experiment in order to guarantee the reproducibility of the manipulation.

### 2.4. Genomic Analysis of the Isolates

The sequences referring to each of the isolates were retrieved from the results previously obtained in order to characterise the isolates. The classification of the isolates was determined using the Nextclade tool (Nextstrain, Nextclade: https://clades.nextstrain.org/ (accessed on 20 May 2021 and 27 November 2021) and the Pangolin classification tool (https://pangolin.cog-uk.io/ (accessed on 20 May 2021), and for the name of the mutation. Mutations leading to structural protein substitutions were grouped with their respective isolates and tabulated. 

The phylogenetic relationship between the different isolates was inferred using the Maximum Likelihood method and the Tamura-Nei model [18]. The heuristic tree was obtained automatically by applying Neighbour-Joining and BioNJ algorithms to a matrix of pairwise distances, which was estimated using the Tamura-Nei model, then by selecting the topology with the higher log likelihood value; the evolutionary analyses were conducted in MEGA X [29].

The network graph presented in this study was built using Gephi version 0.9.2 [30]. The graph components were listed in a common separated values (.csv) spreadsheet, and this file was imported into the software. The layout was generated using algorithms based on the nodes’ forces of attraction and repulsion (Fruchterman Reingold). Modularity class statistics were performed in order to identify and colour the clusters, and the size of the nodes was defined by the gradient incoming. Finally, the nodes were locally rearranged for the better visualisation of the connections between them.

## 3. Results

### 3.1. Viral Replication Analysis

**Figure 1:**
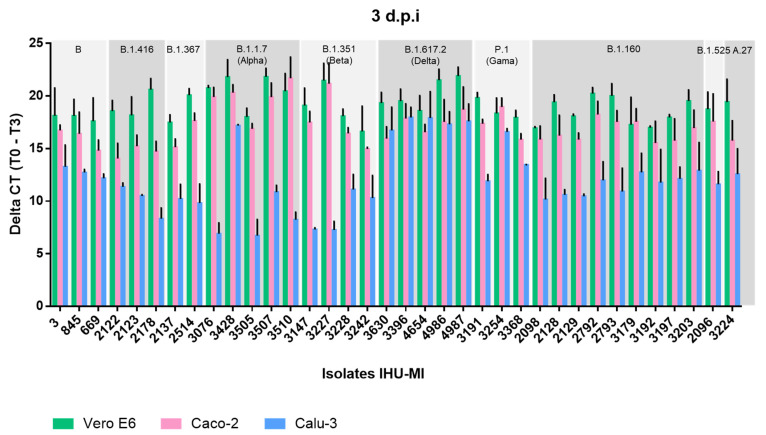
Replication rate of different SARS-CoV-2 lineages isolated in distinct cell lines. The replication rate of 36 SARS-CoV-2 isolates is expressed in delta CT (cycle threshold), three days post-infection, in cells derived from monkey kidney epithelium (Vero E6 in green), human intestines (Caco-2 in pink) and human lungs (Calu-3 in blue). Each isolate was classified according to its lineage, based on the PANGO classification, and viruses from the same lineage were grouped together.

**Figure 2:**
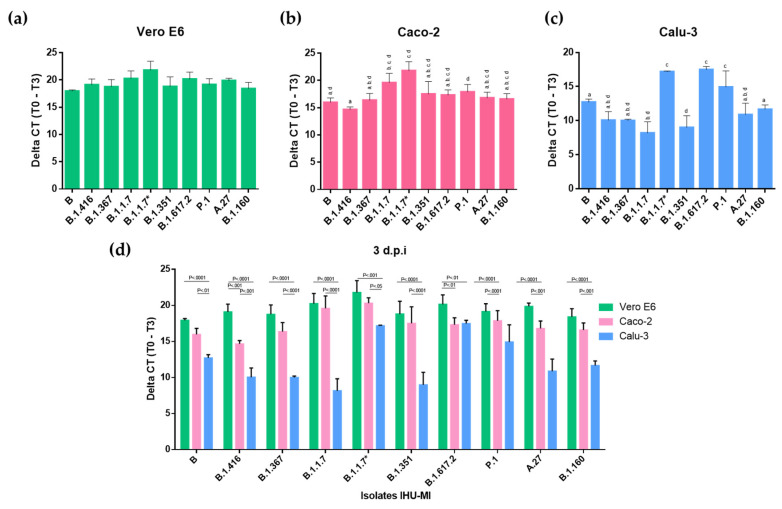
Replication levels of different SARS-CoV-2 lineages in three distinct cell lines. The replication levels of the SARS-CoV-2 lineages (PANGO) are expressed in delta CT (cycle threshold), three days post-infection, in three distinct cell lines: (**a**) Vero E6, (**b**) Caco-2, and (**c**) Calu-3. The values with different superscript letters in a column are significantly different (*p* < 0.05). (**d**) Comparison of the replication of a SARS-CoV-2 isolate from each viral lineage inoculated on different cells: Vero E6 (Green), Caco-2 (Pink) and Calu-3 (Blue).

The replication of different SARS-CoV-2 genotypes was evaluated based on the detection of the viral genome in the supernatant of three different cell lines infected with fresh inoculum from 36 SARS-CoV-2 isolates (Figure 1). Among the viruses inoculated in the Vero E6 cell at an MOI of 0.036, no significant differences were observed between the viral replications of different genotypes, suggesting the good adaptation of all of the viruses to the Vero E6 cells in which the inoculum was produced (Figure 2a). 

During the evaluation of the viral expression in Caco-2 cells (MOI = 0.042), a higher viral production was detected in cells inoculated with SARS-CoV-2 of the B.1.1.7 lineage compared to close Wuhan-Hu-1 relatives of lineage B, and to viruses of lineages B.1.416 and B.1.160 (Figure 1 and Figure 2b). Isolates of the B.1.416 lineage also showed a significantly lower viral replication than those of the P.1 lineage (Figure 2a). However, these isolates did not show significant differences in terms of replication within the same lineage (Figure 1).

In contrast, in Calu-3 (MOI = 0.044), which are lung epithelial cells, there was greater variation in the replication between isolates. In contrast to that which was observed in Caco-2 cells, the most distinct profile was associated with the P.1 and B.1.617.2 genotypes, which presented a higher replication in Calu-3 cells when compared to five of the other eight genotypes tested, which were B, B.1.416, B.1.367, B.1.1.7, B.1.351, A.27 and B.1.160 (Figure 2c). The B.1.1.7 and B.1.351 genotypes presented a lower replication in Calu-3 cells when compared to the virus from lineages B and B.1.160. Exceptionally, isolate IHU-MI3428 (B.1.1.7*) had a high rate of replication in Calu-3 cells, which was significantly higher than the other B.1.1.7, with a replication very similar to that observed for P.1 (Figure 2c).

Interestingly, viruses inoculated on Vero E6 cells and Caco-2 cells presented higher replication when compared to viruses inoculated on Calu-3 (Figure 1 and Figure 2d). While only lineages B.1.416 and B.1.160 showed a significantly lower replication in Caco-2 compared to Vero E6 (Figure 1 and Figure 2d), all of the isolates inoculated in Calu-3 had a lower virus production level. The virus production was also significantly lower when comparing the isolates inoculated in Calu-3 and Caco-2 (Figure 1 and Figure 2d). These comparative results between the viral replications in different cell lines may suggest a greater adaptation of the virus to Vero E6 and Caco-2 cells.

### 3.2. Analysis of Genetic Diversity 

**Figure 3:**
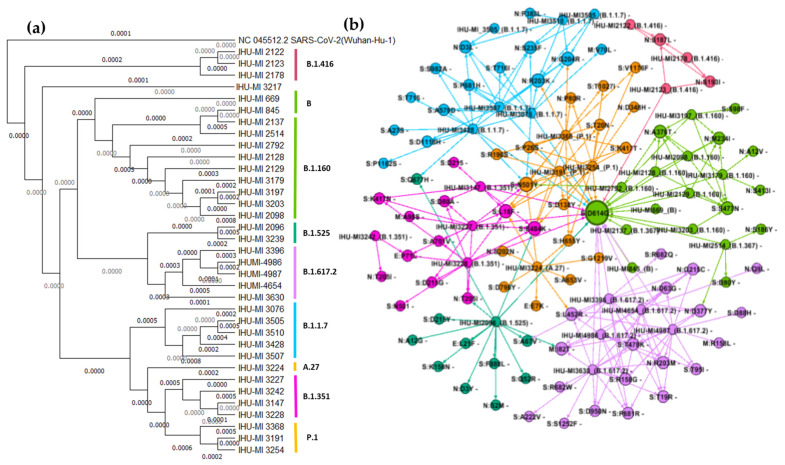
Genetic diversity of the SARS-CoV-2 isolates used. (**a**) Phylogenetic analysis of isolated SARS-CoV-2 presented through a maximum likelihood tree, with a bootstrap analysis based on the values of 1,000 replicates. The isolates were grouped according to the PANGO classification, based on the analysis previously obtained from the Nextclade tool. (**b**) Distribution of the mutations that generate amino acid exchange in structural proteins of SARS-CoV-2 among the isolates used in the study. Arrows link the isolates to their identified mutations; the size of the nodes represents the gradient incoming (the most shared mutations are in bigger circles). Legend: E, envelope protein; M, membrane protein; N, nucleocapsid protein; S, spike protein.

The phylogenetic analysis confirmed that the isolates classified as belonging to the same lineage by the Nextclade tool were close relatives and clustered (Figure 3a). The mutations that resulted in amino acid substitution for each isolate were listed and compared to the initial isolate (b). The close relationship between the isolates observed in the phylogenetic analysis (Figure 3a) was also evidenced by the modular analysis of the mutations conducted by GEPHI to generate the network graphic (Figure 3b). Altogether, 64 mutations in structural proteins were identified in the 36 isolates evaluated in this work, with many of them being shared by several isolates from different lineages. For example, the D614G substitution in the SARS-CoV-2 spike protein was shared by 22 isolates, including the B, B.1.1.7, B.1.160, B.1.351, B.1.367, P.1, and R.1 lineages (Figure 3). Other mutations often shared by viruses of different lineages were N501Y and E484K, both located in the spike protein (Figure 3).

This analysis also revealed isolates that accumulated a variety of unique mutations, such as IHU-MI2096 (B.1.525) and IHU-MI3224 (A.47), with ten and five unique substitutions (not present in any other isolate tested), respectively (Figure 3b).

## 4. Discussion

Considering aspects of the basic virology of SARS-CoV-2, cell culture is a great way of determining the tropism of the virus and its replication level, which are important attributes to be determined in in vitro assays [31]. Many studies have sought to establish cell lines that are permissive to SARS-CoV-2 and different isolates, and they have highlighted Caco-2 and Calu-3 cells as the cells that best support a complete cycle of SARS-CoV-2 replication [2,26,31,32]. 

In this study, we sought to evaluate the replication of different SARS-CoV-2 isolates isolated throughout the pandemic (2020 and 2021) in Marseille, France. For this, we used three distinct cell lines: Vero E6 cells, which originated from monkeys, and Caco-2 and Calu-3 cells, which both originated from human tissues; the replication was assessed by qPCR in the cellular supernatant. 

All of the viruses used in these assays were previously isolated and produced in Vero E6 cells. Vero E6 cells were established from kidney tissue sampled from an African green monkey, and are a mammalian cell line which is widely used for the isolation and production of viruses, including SARS-CoV-2, as these cells abundantly express ACE2 and allow the complete replication cycle of the virus [32,33,34]. 

Even if only a few passages have been conducted in these cells, it was evident that all of the isolated SARS-CoV-2 mutations are well adapted to this cell line, regardless of the lineage to which they belong, as the replication levels in these cells were the same for all of them (Figure 2a). This rapid adaptation was previously reported in trials using SARS-CoV-2 that went through four passages on Vero E6 [34,35]. However, the kinetic analysis of second-passage variants (in Vero E6 cells) pointed out differences in replication between the strains [36].

SARS-CoV-2 replicates in gastrointestinal cells in vivo, and is frequently detected in faeces [37,38,39,40,41]. SARS-CoV-2 RNA has already been detected in samples obtained from gastric mucosa, rectal mucosa, duodenal mucosa and faeces, and therefore the digestive system has been understood as a potential source of transmission [42,43,44]. Caco-2 cells were established from a human colorectal adenocarcinoma [45], and have been widely used to study infection with SARS-CoV [46,47]. SARS-CoV-2 has recently been successfully isolated using Caco-2 cells. Here, we observed that in Caco-2 cells, the lineage B.1.1.7 stood out with a higher viral replication compared to the other lineages, especially to lineage B, which was prevalent in our geographical area in March and April 2020, and to lineage B.1.416 (Figure 2b).

According to the CDC, the lineage B.1.1.7/alpha was initially detected in the United Kingdom, and established itself as a variant of concern [28]. This new variant was attributed to an increased transmission rate of around 50% [19] and a minimal difference in neutralisation by convalescent and post-vaccination sera [11,13,48]. Genomic sequencing revealed 23 mutations [10,48]. Most of the non-synonymous mutations were observed in the gene encoding the spike protein that is responsible for viral adsorption and entry [49].

The impact of these mutations on viral replication, transmission, and pathogenesis is still not well understood, much less the impact of these mutations on the virus replication cycle and cell tropism. A comparison between the replication of one isolate of the B.1.1.7 lineage and one of an early B.1 lineage (containing the D614G substitution) in both Vero E6 and Caco-2 cells did not find any differences in the replication kinetics between these isolates [37]. However, in a study using intestinal organoid models, the B.1.1.7 virus was associated with higher titres of virus production at the end of infection, and with a greater replicative fitness compared to SARS-CoV-2 of lineage B [50]. This supports the evidence that lineage B.1.1.7 would have greater suitability for intestinal cells than the isolates that spread worldwide at the beginning of the pandemic in 2020. 

Calu-3 cells, in turn, are a human lung adenocarcinoma cell line commonly used in cancer research and drug development [51]. In the previous SARS-CoV epidemic, it was initially difficult to establish infection models in cells derived from human lungs, which compromised studies on the pathogenesis of the virus, which mainly causes manifestations in the respiratory tract [52]. Calu-3 cells were then perceived as highly permissive to SARS-CoV infection [52]. These characteristics were also observed for SARS-CoV-2 [2,40]. 

Our results, based on the possession of SARS-CoV-2 regarding the representatives of the diversity of the currently available genotypes, demonstrate that viral replication in Calu-3 cells is the most heterogeneous (Figure 2c). As observed in Caco-2 cells, the B.1.1.7 genotype stood out; however, in Calu-3 cells, its replication was reduced compared to those of isolates of the lineages B, B.1.160 and P.1/gamma (Figure 2c). A similar reduction was observed for variant B.1.351, the replication of which was significantly lower than those for isolates of lineages B and B.1.160 (Figure 2c). 

It was previously described that a B.1.1.7 variant isolate was strongly attenuated on Vero E6 cells compared with a lineage B isolate from the early phase of the pandemic [35,50]. In addition, both B.1.1.7 and early B isolates rapidly acquire multibasic cleavage site (MBCS) mutations on Vero E6 cells, but not on Calu-3 cells [34,50]. Attenuation in Vero E6 cells has also been described for the mink-associated SARS-CoV-2 variant (Cluster 5) [53]. Therefore, passages in Vero E6 cells can lead to the attenuation of isolates of these two lineages, and explain the diminished replication pattern in Calu-3 cells.

On the other hand, the P.1/gamma and B.1.617.2/delta isolates had a higher replication level in Calu-3 cells than viruses from lineages B.1.416, B.1.367, B.1.1.7, B.1.351, and B.1.160. A similar replication pattern was observed for a single isolate belonging to the B.1.1.7 lineage, IHU-MI3428.

The P.1/gamma lineage was initially detected in Brazil [9], and like B.1.1.7, it was considered to be a variant of concern by the CDC [28]. The P.1 variant has ten unique spike protein mutations, including a combination of three important substitutions in the spike protein, E484K and N501Y in its receptor-binding motif, and K417T in its RBD [13]. However, to date, little is known about the transmissibility of the P.1 variant, even though it shares several mutations with the B.1.325 strain (K417N/T, E484K, N501Y), which appears to have increased transmissibility [54]. 

The B.1.617.2 and AY lineages represent the delta variant, which was first detected in India [28] and almost exclusively shares the D614G mutation with P.1/gamma (Figure 3b).The D614G change in the spike protein of SARS-CoV-2 has been suggested to be a factor that enhances the replication and transmission of SARS-CoV-2 [55], and this mutation was present in 29 of the 36 isolates used in our assays (Figure 3), including variants with reduced replication, such as B.1.351/beta. Like P.1/gamma and B.1.617.2/delta, it is also considered a variant of concern associated with increased transmission of approximately 50% [56], and reduced neutralisation by convalescent and post-vaccination sera [20,57,58,59]. 

One study using isogenic variants of SARS-CoV-2 demonstrated that the spike D614G-containing variant has a greater affinity to the ACE2 receptor, increasing replication in primary human bronchi and epithelial nasal airways in vitro. However, no changes were observed to be significant in replication in Vero E6 cells or Calu-3 [11,60]. When the viral titres recovered from washes in hamsters infected with spike D614G SARS-CoV-2 were evaluated, higher infectious titres were recovered in the nasal washes and trachea, but not in the lungs, supporting the clinical evidence that the mutation increases viral loads in the upper respiratory tract of patients with COVID-19 [12,61]. This may explain why, in lung cells, the B.1.1.7 and B.1.351 viruses have reduced expression in pulmonary Calu-3 cells; however, it does not explain the increased replication of the delta, gamma or IHU-MI3428 isolates.

Something similar occurs in the N501Y replacement in the spike protein. SARS-CoV-2 carrying the N501Y substitution exhibited consistent fitness gains in replication in the upper airways in the hamster model and higher fitness at almost all of the time points in Vero and Calu-3 cells [62]. This led to the conclusion that the N501Y substitution improved the affinity of the viral spike for cell receptors. 

However, as noted for D614G, an N501Y substitution is present in P.1, B.1.1.7 (including IHU-MI3428) B.1.351, and A.27. Therefore, it does not explain the different replication patterns between them. Using pseudoviruses with spike proteins designed based on the sequences of viruses of early lineage B and variants B.1.1.7, B.1.351 and P.1, no differences were observed in the pseudovirus entry into Vero E6, Caco-2 and Calu-3 cells [63].

When comparing virus production by different cells, it seems usual for Vero E6 and Caco-2 cells to have better viral production than Calu-3 [1,33,64,65,66]. Interestingly, in a previous study that evaluated 13 human cell lines that could potentially support the replication of the first SARS-CoV, Caco-2 cells were the only cell line found that allowed the complete virus cycle and were shown to be as efficient as African green monkey cells [67]. One more recent study reports that SARS-CoV infects and replicates more efficiently in Caco-2 cells than in Calu-3 cells under the same MOI [2]. Thus, the results in which Vero E6 cells and Caco-2 cells have comparable replication levels are consistent with what was previously reported, while Calu-3 cells produce less SARS-CoV-2 compared to these two cells (Figure 2d) [65,66,67]. This was also reported for patients in a study suggesting that viral shedding from the digestive tract might be greater than that from the respiratory tract [41,42]. 

Despite the similarity in the present study with the increase of replication in Calu-3 of isolates from the gamma and delta variants, IHU-MI3428 (B.1.1.7*) does not seem to have the same mutations as a source, as they share only the N501Y and D614G mutations, which are widely distributed among the isolates (Figure 3). The greater replication of these isolates in Calu-3 cells must be associated with a unique combination of mutations found in these isolates. 

The P.1 isolates share several mutations in the spike protein restricted to this variant, according to our comparative analysis between the isolates used in this study (Figure 3). The IHUMI-3428 (B.1.1.7) isolate presents other interesting mutations, such as Q677H in the spike protein, which is absent in the other B.1.1.7, with reduced replication (Figure 3). This mutation would have appeared independently in several strains in the USA, in an adaptation suggested due to the alterations associated with this mutation in the proximal polybasic furin cleavage site [68,69]. In addition to the Q677H mutation, IHU-MI3428 has two other unique mutations in the spike protein, A27S and P1162S (Figure 3). However, Q677H is shared with another isolate, IHU-MI2096 (B.1.160), which does not seem to have increased replication, reinforcing the evidence that it is the complex combination of mutations that makes certain isolates better adapted to replication in specific cells.

## 5. Conclusions

The current genome diversity of SARS-CoV-2 has a clear impact on clinical practice, especially regarding transmission rates and the effectiveness of vaccines, making it difficult to effectively combat COVID-19. However, it also poses challenges to basic virology and the standardisation of in vitro assays, as evidenced by the differences in the replication levels for each SARS-CoV-2 lineage in different human cells. Variants such as alpha, beta, delta and gamma, for example, can show distinct replication patterns if they are inoculated into cells derived from the lungs or intestines compared to a clade B lineage, which was responsible for the first wave of the pandemic in early 2020, and which was the focus of many of the basic virology/in vitro studies. These variations in replication patterns among SARS-CoV-2 isolates appear to result from the complex combination of mutations that make certain isolates better adapted to replication in specific cells. These results underscore the importance for all of the research groups working with SARS-CoV-2 to evaluate and characterise their SARS-CoV-2 isolates to establish the replication patterns before performing their tests, and to consider the optimal combination of viral genotype and cell type for these assays.

## Data Availability

The genome sequences were submitted to the GISAID database (https://www.gisaid.org/ (accessed on 20 May 2021).

## Supplementary Materials

The following are available online at https://www.mdpi.com/article/10.3390/v14010023/s1—Table S1: List of isolated SARS-CoV-2 lineages and their respective genotypes.
