# Supplementary material for: Emerging SARS-CoV-2 Genotypes Show Different Replication Patterns in Human Pulmonary and Intestinal Epithelial Cells
Source: Viruses. 2021 Dec 23;14(1):23. doi: 10.3390/v14010023 (PMC8777977; doi:10.3390/v14010023)
Supplement: Supplementary file 1 [file viruses-14-00023-s001.zip › Table S1 - R1.pdf]

Table S1. List of isolated SARS-CoV-2 and their respective genotypes

	IHU-MI Isolates	Lineage (PANGO)	WHO Label	CDC Classification	Country first detected (community)
1	3				
2	669	B	–	–	China
3	845				
4	2122				
5	2123	B.1.416	–	–	Senegal/Gambia
6	2178				
7	2137	B.1.367	–	–	Europe (multiple countries)
8	2514				
9	3076				
10	3428				
11	3505	B.1.1.7	Alpha	Variant of Concern	United Kingdom
12	3507				
13	3510				
14	3147				
15	3227	B.1.351	Beta	Variant of Concern	South Africa
16	3228				
17	3242				
18	3396				
19	3630				
20	4654	B.1.617.2	Delta	Variant of Concern	India
21	4986				
22	4987				
23	3191				
24	3254	P.1	Gamma	Variant of Concern	Brazil
25	3368				
26	2098				
27	2128				
28	2129				
29	2792				
30	2793	B.1.160	-	-	Europe and United Kingdom (multiple countries)
31	3179				
32	3192				
33	3197				
34	3203				
35	2096	B.1.525	Eta	Variants Being Monitored	United Kingdom and Nigeria (multiple countries)
36	3224	A.27	-	-	Unclear
